# Fluro-Protein C-Phycocyanin Docked Silver Nanocomposite Accelerates Cell Migration through NFĸB Signaling Pathway

**DOI:** 10.3390/ijms24043184

**Published:** 2023-02-06

**Authors:** Harishkumar Madhyastha, Radha Madhyastha, Eshika Chakraborty, Kaushita Banerjee, Kamal Shah, Yuichi Nakajima, Nagendra Singh Chauhan, Sajitha Lulu Sudhakaran, Kaoru Ohe, Gothandam Kodiveri Muthukaliannan, Abilash Valsala Gopalakrishnan, Masugi Maruyama, Nozomi Watanabe

**Affiliations:** 1 Division of Cardiovascular Physiology, Faculty of Medicine, University of Miyazaki, Miyazaki 8891692, Japan; 2 School of Biosciences and Technology, Vellore Institute of Technology University, Vellore 632014, India; 3 Institute of Pharmaceutical Research, GLA University, Mathura 281406, India; 4 Drug Testing Laboratory, Avam Anusandhan Kendra, Raipur 490008, India; 5 Department of Applied Chemistry, Faculty of Engineering, University of Miyazaki, Miyazaki 8892192, Japan

**Keywords:** silver doped C-phycocyanin (AgcPCNP), cell migration, cell proliferation, fibroblast cells, NFĸB pathway, wound healing

## Abstract

Currently, there is a great demand for the development of nanomedicine aided wound tissue regeneration via silver doped nanoceuticals. Unfortunately, very little research is being carried out on antioxidants-doped silver nanometals and their interaction on the signaling axis during the bio-interface mechanism. In this study, c-phycocyanin primed silver nano hybrids (AgcPCNP) were prepared and analyzed for properties such as cytotoxicity, metal decay, nanoconjugate stability, size expansion, and antioxidant features. Fluctuations in the expression of marker genes during cell migration phenomena in in vitro wound healing scenarios were also validated. Studies revealed that physiologically relevant ionic solutions did not exhibit any adverse effects on the nanoconjugate stability. However, acidic, alkali, and ethanol solutions completely denatured the AgcPCNP conjugates. Signal transduction RT^2^PCR array demonstrated that genes associated with NFĸB- and PI3K-pathways were significantly (*p* < 0.5%) altered between AgcPCNP and AgNP groups. Specific inhibitors of NFĸB (Nfi) and PI3K (LY294002) pathways confirmed the involvement of NFĸB signaling axes. In vitro wound healing assay demonstrated that NFĸB pathway plays a prime role in the fibroblast cell migration. In conclusion, the present investigation revealed that surface functionalized AgcPCNP accelerated the fibroblast cell migration and can be further explored for wound healing biomedical applications.

## 1. Introduction

Biological applications of silver and silver salts have been in practice in the areas of agriculture, traditional medicine, and microbiology since the genesis of human civilization. However, their materialistic application to regenerative medicine is of recent origin. Silver nanoparticle (AgNP) adjuvants are now being used in various biomedical and consumable products. The unique properties of AgNPs have enabled their extensive use in various fields including industrial, household, healthcare, the food industry, diagnostics, biomedical gadgets, cosmetics, and medicine. They are synthesized by physical, chemical and biological methods, using two approaches, namely “bottom-up” and “top-down” processes [1]. In the biological route, green synthesis has attained considerable attention for its scalable non-toxic application towards human use. Biomedical and biological applications of AgNPs are directly linked to various external factors such as surface chemistry, shape, size, distribution of different particles in colloidal solution, morphology, capping of surface refined material, the efficacy of active ion release, and the nature of reducing and stabilizing agents used during the synthesis process [2]. The natural property of silver to inhibit the nucleic acid and protein synthesis machinery of microorganisms, eventually leading to their death, finds immense value in regenerative medicine.

Bio and inert material assisted wound healing is a modern regenerative therapy aspect in the present day [3,4]. Various biomaterials for better wound management abilities have been attempted for better tissue growth [5]. Nano materials can be genuine curative materials for chronic wound complications. Microorganisms in the wound bed micro-niche delay the healing pattern and also hamper the health status of the different cell types involved in the process of wound healing. Complex physico-chemo-biological events result in the formation of biofilm at the wound bed and further hamper the hemostatic phenomenon of the wound healing [6]. Systematic antibiotic-loaded topical wound healing materials can rarely penetrate the biofilm, resulting in wound sensitization and septic conditions. Although surgical debridement of the wound bed can be effective in controlling the bacterial population, the bacterial recalcitrance to antibiotics shields the drug resistant bacteria that persist in the wound bed. The judicious and selective use of silver as an adjuvant material in wound care situations can accelerate the healing process but does not provide the complete benefit from delayed healing. Nano-silver conjugates have emerged as prominent alternatives in the resolution of third-degree non-healing wounds [7,8]. It is reported that AgNPs can block the respiratory signaling track of fibroblasts and keratinocytes, and help in the migration, proliferation and remodeling of the cells [9]. Recent nanocomposts developed for chronic wound management incorporate various nutraceutical compounds with silver for various beneficial aspects, such as anti-oxidant, anti-inflammatory, and surface modifiers properties and also to prevent the inherent toxic phenomenon of silver metal to native wound healing marker cells such as fibroblasts and keratinocytes [8,10,11,12]. Several nanotechnologies have been standardized and commercialized to address the specific area of dermal tissue repair, especially diabetic wounds. C-Phycocyanin (cPC) is one such well-established and FDA-approved cyanobacterial metabolite material found to have applications in wound healing [7,13]. Previously, we prepared cPC coronated silver nanoparticles (AgcPCNPs) and demonstrated that they facilitate blood cell resilience mechanisms and also help in wound healing [7]. In this study, we evaluated the mechanistic aspects of wound healing along with the possible use of AgcPCNP as a potential wound healing agent with an insight into various signaling regulations during the in vitro wound healing process. An RT^2^PCR array is a reliable tool for the focused analysis of marker genes in various signaling networks. Different signaling pathways play important roles in dermal wound healing. For example, NRF2 activation helps in cell proliferation, neovascularization, and repair of damaged tissues [14]. Activated Wnt signaling helps in programmed cell death, chemoattractions of therapeutic drugs, the differentiation of dermal stem cells and the control of inflammation during wound healing [15]. Tissue redox signaling is a key regulator of wound healing by maintaining the oxygen hemostasis through the scavenging of reactive oxygen species in early stages [16,17]. The intriguing connection between eicosanoids and phospholipids during dermal wound healing is carefully governed by lipid signaling pathways [18]. Multifunctional growth factor TGF-β/SMAD has a prominent role in wound closure, contraction, and re-epithelization that occurs during wound healing [19]. At the inflammatory stage of wound healing, there is a fine line between the NFĸB pathway and inflammatory cytokines, which may serve as potential targets, ultimately leading to different pathophysiological events [20].

This study documents the molecular mechanism and cross talks between different signaling cascades during the AgcPCNP assisted fibroblast cell migration and in vitro wound healing process.

## 2. Results and Discussion

The use of non-toxic biological compounds produced by simple and efficient oxidation reactions is considered as a smart and viable choice over the complex chemical and physical methods in the synthesis of nanoparticles for biomedical use. This process is defined as green synthesis [21]. Fluro-protein c-Phycocyanin (cPC) from cyanobacteria *Spirulina fusiform* has numerous proved applications in medicine, nutrition, biotechnology, nanotechnology and pharmaceuticals with an anti-oxidant, anti-inflammatory, anti-cancer, pro-immunomodulatory and healing capacity of certain skin ailments and injuries [7,13,22,23]. Initially, we predicted and confirmed the docking position of AgNP with cPC. The 3D co-ordinates of cPC were retrieved from the Protein Data Bank (PDB ID: 1CPC). The target protein, cPC, was modelled with silver ion co-ordinates obtained from the template protein ferritin (PDB ID: 2X17), which is expressed in *Pyrococcus furioscus*. The modelling was executed using the Matchmaker tool Chimera, which operates by generating pairwise sequence alignments between target and template protein sequences. The sequences of target and template proteins were aligned and superimposed using the Matchmaker tool Chimera. The protein chains of the template sequence were deleted by retaining the co-ordinates of silver ions with the target sequence.

**Figure 1:**
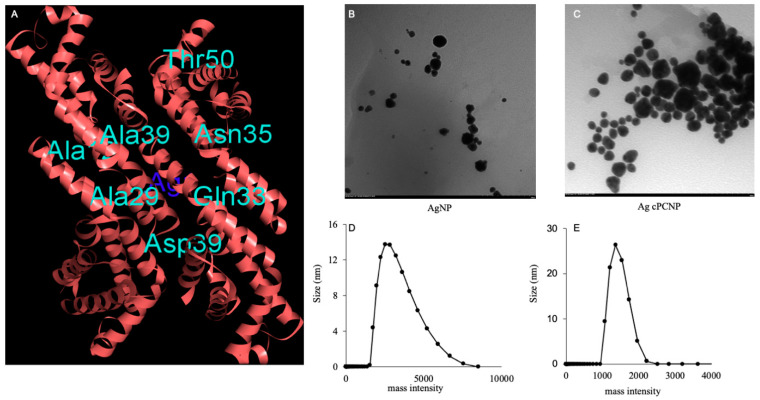
C-Phycocyanin and Silver metal interaction study as revealed by bioinformatic studies (**A**). TEM images depict the morphology and shape of AgNP and AgcPCNP (magnification ×40.0 K) (**B**,**C**). Size analysis by DLS studies of AgNP and AgcPCNP in nm (**D**,**E**).

**Figure 2:**
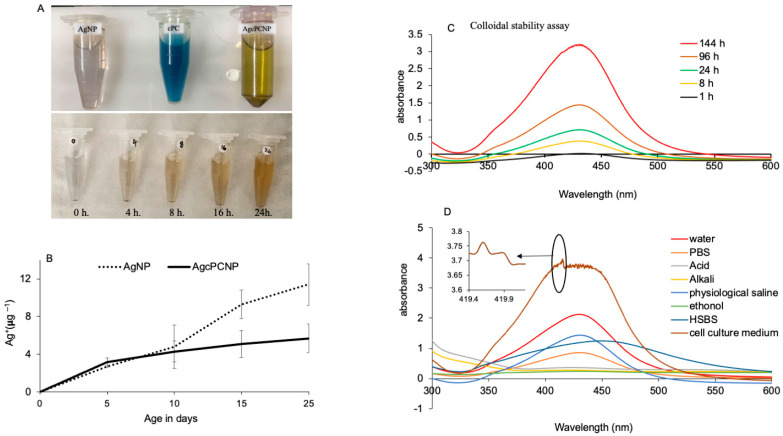
Color intensity changes during the different reduction incubation times in hr (**A**). Leaching of Ag^+^ per μg L^−1^ from AgNP and AgcPCNP over different days (**B**). Colloidal stability assay at different time periods from 1 h to 144 h (**C**). Stability of NPs in different solutions (**D**).

The cPC-silver model was visualized in Discovery Studio. The amino acid residues residing in the proximal region of Ag atoms included Thr50, Ala39, Asn35, Ala29, Gln33 and Asp39 (Figure 1A). The cPC silver conjugate was prepared following the simple environmentally envisaged method, and the physico-chemical characterization was published [7]. Morphological analysis by TEM analysis revealed the clear spherical shape (Figure 1B,C). DLS analysis revealed the size of AgNPs and AgcPCNPs to be about13 nm and 27 nm, respectively (Figure 1D,E). The larger size of AgcPCNPs demonstrates the successful corona formation of cPC on silver metal and confirms our previous studies [7]. The reduction kinetics of silver ion to silver particles is evident from the color change of AgNO_3_ from pale to dark brown after the addition of 75 µg mL^−1^ cPC over a 24 h incubation time (Figure 2A). The phenomenon of color change is explained by the principle of surface plasma resonance [24] and the excitation of outer surface electrons. A surface plasma resonance peak at 407 nm further confirmed the formation of AgcPCNP.

The leaching of Ag^+^ from AgNP and AgcPCNP over 25 days was analyzed by quantifying the amount of Ag metal in the solution by ICPS. The rate of Ag^+^ leaching from AgNPs and AgcPCNPs was similar for 10 days; later on, a rapid increase of Ag^+^ release was observed in AgNPs in contrast to a minimal insignificant release from AgcPCNPs (Figure 2B). This indicated that cPC helped as a stabilizing agent during the process of NP synthesis, and aided in colloidal stability. The time dependent kinetics of the colloidal stability of AgcPCNP were analyzed by the UV-VIS method at different periods of 1, 8, 24, 96 and 144 h, and with different solvents such as de-ionised water, phosphate-buffered saline (PBS), mild acid (0.1 M HCl), mild alkali (0.1 M NaOH), physiological saline (0.9% Nacl W/V), ethanol (100%), Hanks balanced salt solution (HSBS) and cell culture medium. λ_max_ was noticed at 407 nm in all time-tested periods, confirming that AgcPCNP was stable at room temperature (Figure 2C). Incubation with different solvents also projected similar results, with a maximum absorbance of 407 nm (Figure 2D). The additional shoulder peak at 402 nm obtained with the cell culture medium indicated probable surface interactions of AgcPCNP with the cell culture medium metabolites as a capping phenomenon.

**Figure 3:**
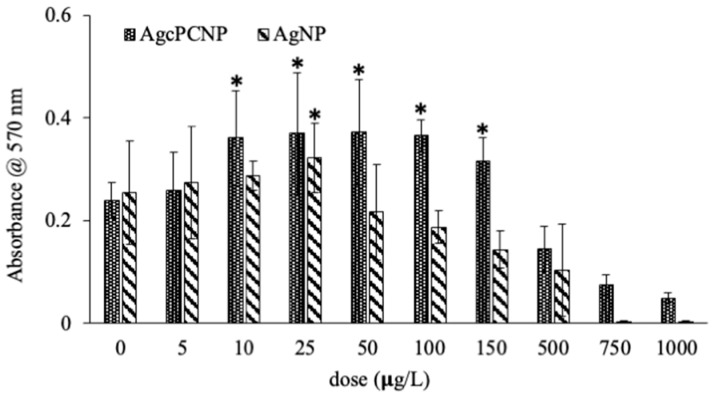
Dose-dependent (0 μg L^−1^ to 1 mg L^−1^ of Ag) cytotoxicity assay of AgNP and AgcPCNP. Doses of Ag metal concentrations are calibrated to μg L^−1^. All experiments were conducted in three independent sets and *p* < 0.05% was set as the significant level between each group (* *p* < 0.05).

The degree of nanometal toxicity in tissues and cells is of paramount importance for biomedical applications for use in humans [12]. In the present investigation, an end point toxicity assay revealed dose-dependent activity of AgNP and AgcPCNP (Figure 3).

**Figure 4:**
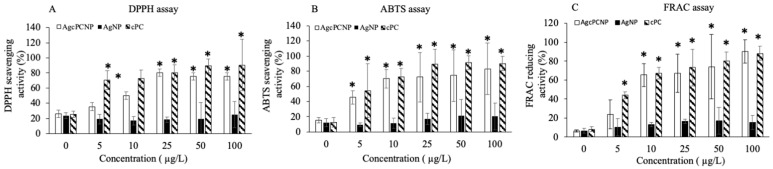
DPPH (**A**), ABTS (**B**), and FRAC (**C**) antioxidant assay. Antioxidant efficacy of AgNP, AgcPCNP and cPC was conducted with varying concentration of 0, 5, 10, 25, 50 and 100 μg L^−1^. All experiments were conducted in three independent sets and *p* < 0.05% was set as the significant level between each group and compared with the untreated control group (* *p* < 0.05).

**Figure 5:**
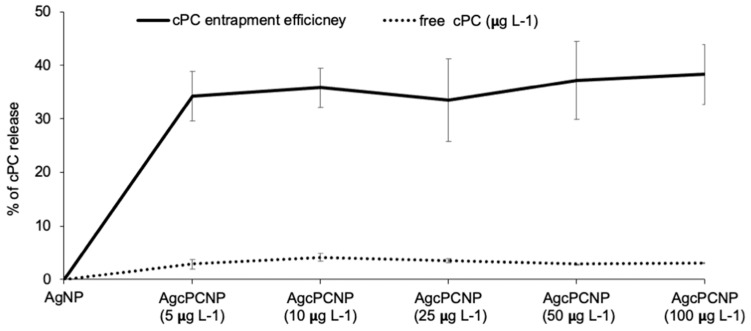
cPC entrapment assay. Amount of cPC leached from different AgcPCNP concentrations (5 μg L^−1^, 10 μg L^−1^, 25 μg L^−1^, 50 g L^−1^ and 100 μg L^−1^) was carried out at constant incubation time of 24 h. Free cPC amount was analyzed by spectrophotometry (λ_max_ 560 nm). Percentage of cPC released was calculated by using standard formula. All measurements were conducted in triplicate and data are expressed as mean ± standard deviation (*p* < 0.05).

AgcPCNP demonstrated better activity than the AgNP. This observation is in conformation with earlier reports which revealed that nanometal formulations that are surface functionalized by antioxidant compounds show better biological activity and are considered as advantageous analogues to combat metal toxicity in cellular mileu [7,25]. Oxidative stress linked cellular damage is a hallmark of many pathophysiological events such as ageing, diabetes, cardiovascular diseases, chronic inflammation, atherosclerosis, delayed wound healing, and cancer [26]. In the present study, the in vitro antioxidant activity of AgNP, AgcPCNP and native cPC was examined by DPPH (Figure 4A), ABTS (Figure 4B), and FRAP (Figure 4C) scavenging assays. AgNP exhibited a negligible amount of antioxidant properties in DPPH (18.2–20.2%), ABTS (7.2–16.3%) and FRAC (5.6–13.2%) in different concentrations tested; on the other hand, AgcPCNP demonstrated better antioxidant scavenging activity. The better antioxidant scavenging activity of AgcPCNP against DPPH and ABTS radicals can be attributed to free radical quenching via H^+^ transmission due to the presence of cPC, which is a proven singlet oxygen quencher. The tetrapyrrole structure of cPC moiety further helped to scavenge the FRAP, as observed in Figure 4C, where cPC and AgcPCNP showed the highest percentage of scavenging activity at 100 μg L^−1^ concentration. We next analyzed the cPC entrapment coefficient in different compositions of AgcPCNP (5, 10, 25, 50, and 100 μg L^−1^). The amount of free cPC varied from 32.2 ± 1.3 % to 35.6 ± 6.2% in AgcPCNP conjugates (Figure 5), confirming the efficient entrapment of cPC with AgNP.

Antioxidant coronized metal nanoparticles are an emerging class of material for use in various biomedical applications and regenerative adjuvants [7,27,28,29]. Several nanotechnologies that address the specific area of dermal engineering especially for diabetic wounds have been standardized and commercialized. However, molecular mechanisms and crosstalk between different signaling cascades are poorly documented. In order to address this lacuna, it is important to study the interaction of different signaling molecules that are cooperatively and heterogeneously expressed and/or activated during drug stimulation. Different intracellular signaling cascades such as NFĸB, PI3K/Akt, MAPK, PKC/PKR and TLR/RIG-1 pathways play governing roles in dermal wound healing [30]. Here, we used an RT^2^ PCR array to study a set of primary signal transduction genes. Major signaling pathways, which are associated with cell proliferation and development during wound healing, are tabulated in Table 1. Additionally, important marker genes associated with each pathway are depicted in Table 2, Table 3 and Table 4. This array technique was implemented to detect the expressions patterns of the genes in control, AgNP and AgcPCNP treated groups. Data analysis revealed that genes related to the NFĸB and PI3K pathways were significantly altered in AgNP and AgcPCNP groups in comparison to the control group (Table 2, Table 3 and Table 4). However, marker genes in other pathways did not show significant fluctuations between groups.

**Figure 6:**
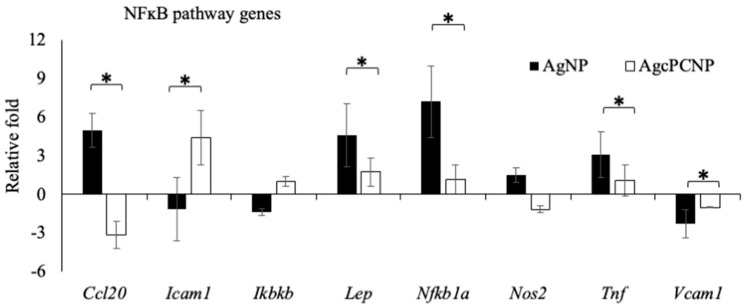
Graphical representation of marker gene fluctuations of NFĸB signaling pathways. All experiments were conducted in three independent sets and * *p* < 0.05 was set as the significant level between each group.

Among the NFĸB pathway members, a significant (*p* < 0.5%) down regulation in the expression levels of *Ccl20, Lep, Tnf, NFĸb1a* and *Vcam1* genes and overexpression of Icam1 were observed in the AgcPCNP group in comparison to the AgNP group (Figure 6). Among the panel of six marker genes of the PI3K pathway (*Bcl2, Ccnd1, Fnf1, Jun, Mmp7* and *Myc*), *Fnf1* and *Mmp7* genes were significantly upregulated in the AgcPCNP group over the AgNP group. This indicates the regulation of NFĸB and PI3K signaling pathways by AgcPCNP. NFĸB and PI3K pathways have prominent roles in different phases of wound healing. NFĸB is a conserved nuclear transcription factor vital for the wound healing process. PI3K is an important signaling junction pivotal for fibroblast cell migration [17].

**Figure 7:**
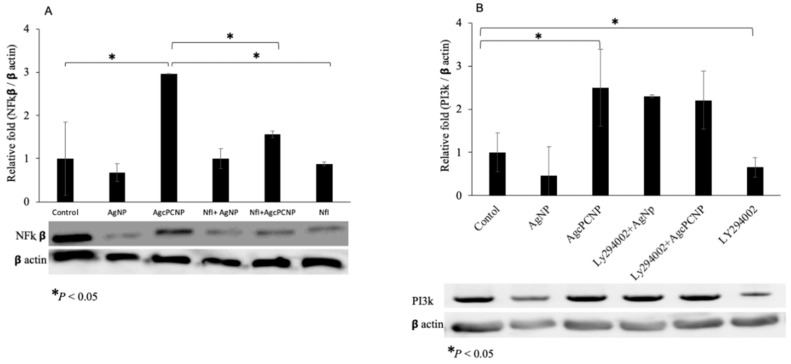
NFĸB (**A**) and PI3K (**B**) protein expression patterns as revealed by Western blot analysis. Cells were treated with or without Nfi (NFĸB pathway inhibitor) and Ly294002 (PI3K pathway inhibitor), along with AgNP and AgcPCNP groups. βactin was used as a loading control. Relative fold expressions were calculated in each experimental set and * *p* < 0.05% was set as the significant level between each group and compared with the untreated control group.

**Figure 8:**
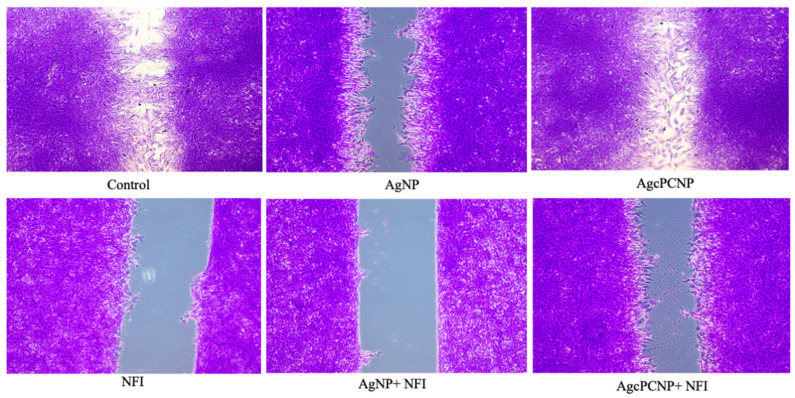
In vitro cell migration assay. Patterns of cell migration were visualized during treatment with AgNP and AgcPCNP along with NFĸB inhibitor (Nfi).

We proceeded to study whether AgcPCNP can modulate wound healing through NFĸB and PI3K pathways by employing specific inhibitors for NFĸB (Nfi, 481407 Insolution) and PI3K (LY2940002). The results of pathway inhibition studies are depicted in Figure 7A,B). Co-stimulation by AgcPCNP or AgNP with respective inhibitors revealed the significant downregulation of the NFĸB protein, but not PI3K, indicating the relevance of NFĸB-dependent activity during the fibroblast in vitro cell migration. Wound healing scratch assay results further confirmed the role of NFĸB as an important signaling cascade junction for AgcPCNP-aided wound healing (Figure 8).

## 3. Materials and Method

### 3.1. Chemicals and Materials

Silver nitrate (AgNO_3_), trisodium citrate (Na_3_C_6_H_5_O_7_), 30 KDa cellulose dialysis membrane (Viskase sales Corp. Tokyo, Japan), FBS and PSN were purchased from Sigma-Aldrich Chemical Co. (St. Louis, MO, USA). c-Phycocyanin (c-PC) dry powder (>95% pure) was a kind gift from Parry Nutraceutical Ltd. Chennai, India. HSBS media was purchased from (Gibco Thermo-Fisher. Co., Ltd., Waltham, MA, USA). All other analytical grade chemicals were purchased from Wako Pure Chemical (Wako Chemicals, Tokyo, Japan). All solutions were prepared with deionized MilliQ water (Millipore-Direct-Q^®^ 3 UV, Millipore, MA, USA).

### 3.2. Cell Culture and Experimental Design

m5S mouse skin fibroblasts (RIKEN Cell bank, Ibaraki, Japan) were cultured in αMEM medium according to standard conditions in a serum free condition. AgNPs and AgcPCNPs prepared according to a standard ratio of silver metals (µg L^−1^) [7] were tested for various experiments as mentioned below.

### 3.3. Bio-Informatic and Molecular Docking Analysis

Interactions of AgNP and cPC were predicted by using match-making chimera systems, which use the computational modelling system as per the guidelines of Matchmaking@GLBIO session, # GenoMatch, #CompMatchBio [31].

### 3.4. Characterization of AgcPCNP (TEM, DLS, ICPS)

In our previous studies, we standardized the methodology of thermoregulated synthesis and physical characterization of AgNP and AgcPCNPs [7]. The spectral properties of AgNPs and AgcPCNPs were measured by UV-Vis Nano Drop spectrophotometer with λ_max_ of 417 nm (ND 1000, Nano Drop technologies, Inc., Wilmington, NC, USA). The morphology was measured by using an ultra-high-resolution transmission electron microscope (200 kV, HT-7700, Hitachi, Tokyo, Japan), wherein a drop of the sample (~5 μL) placed on a carbon-coated Cu grid was dried under infrared light before analysis. The particle size distribution was measured at 25 °C with a light scattering instrument (Zettaliter Nano ZS, Malvern Panalytical, Malvern, UK).

### 3.5. End Point Toxicity Assay

The 4 × 10^4^ cells/mL m5S fibroblasts (third passage) were cultured in αMEM conditioned with 10% FBS and 1% PSN antibacterial cocktail (Nacalai Tesque Inc., Tokyo, Japan) at 37 °C, 5% CO_2_ and 95% humidity. Fibroblast cells were treated with different doses of AgNPs and AgcPCNPs (0, 0.5, 10, 25, 50, 100, 150, 500, 750 and 1000 µg L^−1^) for a fixed time of 16 h. The percentage of viable cells was analyzed by standard procedure of MTT [32]. Intracellular purple formazan was quantified with spectrophotometer at absorbance of 570 nm (Multiskan FC, Thermo Fisher Scientific Inc., Pittsburg, PA, USA).

### 3.6. Radical Scavenging Assay

Free-radical scavenging efficiency of the AgNPs and AgcPCNPs was analyzed by the DPPH reduction method by using commercial kits (Dojindo Laboratories, Kumamoto, Japan). Different doses of AgNP or AgcPCNP (0, 0.5, 1.0, 25, 50 and 100 µg L^−1^) were mixed with DPPH solution and absorbance was measured at 570 nm (Multiskan FC, Thermo Fisher Scientific Inc., Pittsburg, PA, USA). Change in the color of DPPH from violet to yellow was used as an index of free-radical scavenging efficacy of the NPs. Results are presented as the mean value of nine determinations. The percentage of radical scavenging was calculated by using the following formula:

(1) $$\mathrm{Radical}\;\mathrm{scavenging}\;\%=100\;\frac{A_{\mathrm{control}}-A_{\mathrm{sample}}}{A_{\mathrm{control}}}$$

(A_control_: absorbance of control DPPH solution; A_sample_: absorbance of the sample Au^Qur^NPs mixed with DPPH solution).

Commercial kits were used for ABTS assay (CS0790, Sigma-Aldrich Co., Ltd., St. Louis, MO, USA) and FRAC assay (STA-859, Cell Biolabs, Inc., San Diego, CA, USA).

### 3.7. c-Phycocyanin Entrapment Assay

Conjugated AgcPCNP was mixed in MilliQ water (V/V) and sonicated (500 htz/s) at 37 °C for 5 min (Power Sonic, Co., Ltd., Hwashin technology, Seoul, Republic of Korea). Supernatant was collected and the released cPC was measured by spectrophotometer (Shimadzu UV/VIS spectrophotometer, Tokyo, Japan) at a wavelength of 692 nm. All measurements were conducted in triplicate and data were expressed as mean ± standard deviation. cPC encapsulation efficiency was calculated according to a standard formula of regression analysis by using standard phycocyanin.

% Encapsulation Efficiency = (Total amount of phycocyanin added − Amount of free phycocyanin supernatant/total amount of phycocyanin added) × 100.

### 3.8. Nanoparticle Ageing and Stability Assay

Ageing interns of silver ion (Ag^+^) release from AgNP and AgcPCNP nanoconjugates was measured in a time sequence period of different days (0, 5, 10, 15 and 25 days). Initially, AgNPs and AgcPCNPs were suspended in LC-MS pure water and digested with ultra-pure HNO_3_ and 30% hydrogen peroxide (H_2_O_2_) at 115 °C for 30 min. The resultant mixtures were further subjected to integrated coupled plasma spectroscopy (ICPS) following standard procedures. The ion mass of current to previous days was calculated to obtain the amount of Ag^+^ release to medium and measured instrumentally (ICPS-7510, Shimadzu, Tokyo, Japan).

### 3.9. Colloidal Stability Assay

The colloidal nanoparticle stability test was calculated by measuring the changes in λ_max_ at 420 nm. Interactions of AgcPCNPs with different physiologically relevant solvents, such as MilliQ water, PBS, 0.1 mM HCl, 0.1 mM NaOH, physiological saline, 50% (V/V) ethanol, HSBS and cell culture medium with 10% FBS (α MEM), were evaluated by using standard procedures [33]. A standard incubation time of 12 h at 37 °C was followed throughout. Absorbance was measured by UV-VIS spectroscopy (UV 1601, Shimadzu, Tokyo, Japan) between wavelengths ranging from 300 nm to 600 nm.

### 3.10. RT^2^ Profiler Signal Transduction Pathway Finder PCR Array

Third passage m5S fibroblast cells (4 × 10^4^ cells/mL) were cultured in αMEM conditioned with 10% FBS and 1% antibacterial cocktail at 37 °C, 5% CO_2_ and 95% humidity conditioned cell culture incubator. Confluent cells were treated with a single biocompatible dose of 10 µg L^−1^ AgNP or AgcPCNP for 12 h duration. Untreated cells served as a control group. Total RNA was isolated, and first strand cDNA was synthesized using 100 ng of total RNA. An RT^2^-profiler PCR array mouse signal transduction pathway finder kit (S A Biosciences/ Qiagen, Frederick, MD, USA) was used to study the fluctuations of various genes involved in signal transduction. The selected PCR array is a pre-defined set of 88 genes involved in various signal transduction pathways. RT^2^-PCR array was performed using ABI Prism^®^ 7500 HT sequence detection system (Applied Biosystems, Foster city, CA, USA) in the presence of SYBR Green/ROX master mix (SA Biosciences) as per manufacturers’ instructions. Gene profiling and data analysis were performed using array software (SA Biosciences), using the comparative threshold cycle (CT) method to determine the relative expression levels of mRNA of interest in each stimulated group as well as control groups. An average of three independent assays was obtained to compare the actual variations of expressions from stochastic fluctuations.

### 3.11. Immunoblot Assay

m5S cells were cultured in a 6 well culture dish and incubated for 16 h with AgNP or AgcPCNP (10 µg L^−1^), in the presence of either Nfi (NFĸB activation inhibitor; 10 nm) or LY294002 (PI3K pathway inhibitor; 5 nm). Treated cells were washed with cold PBS and lysed using RIPA buffer with 0.5% protease cocktail (Nacalai, Tesque, Inc., Tokyo, Japan). The 10 µg protein samples were electrophoresed over 10% SDS-PAGE gels and electroblotted onto PVDF membrane using Trans-blot SD semi dry transfer cell (BioRad laboratories, Hercules, CA, USA). Membranes were blocked with 5% (w/v) fetal bovine serum albumin and incubated overnight with specific primary antibodies: mouse monoclonal antibodies for NFĸB (1:1000 dilution) or PI3K (1:2000 dilution). Rabbit monoclonal antibody for βactin (Cell signaling technology, Danvers, MO, USA) was used as a loading control. After standard procedures of washing, membranes were incubated with HRP-conjugated secondary antibodies for 1 h at room temperature. The expression of proteins was detected by chemiluminescence using the ECL Plus Western blotting detection system (Amersham Life Science, Inc., Buckinghamshire, UK). The intensities of the protein bands were quantified with the digital imaging system (LAS 4000, Fujifilm, Tokyo, Japan) and images were quantified using band intensities by using Image Quant TL Software (GE Healthcare, Tokyo, Japan).

### 3.12. In Vitro Wound Healing Assay

In vitro wound healing assay was performed using m5S fibroblasts grown in µ-dish culture inserts (Ibidi Suppliers, Lochhamer, Grafelfing, Germany). Cells were cultured in culture vessels supported with µ-dish culture insert to confluency level. At the confluent stage, culture inserts were gently removed without disturbing the edge, to mimic the wound. Cells were treated with 10 µgL^−1^ AgNPs or AgcPCNPs, in the presence of Nfi, and photographed at various time periods (0, 12 and 24 h). The rate of cell migration (mm) in various groups was calculated by using Image J software (NIH, Bethesda, Maryland, USA).

### 3.13. Statistical Analysis

Results were expressed as mean ± standard deviation of control and treated cells from three independent experiments. Nine replicates were first subjected to basic distributive statistics to judge the pattern of normal distribution. After judging the distribution pattern, further statistical analysis was carried out using a non-parametric test using the Kruskal–Wallis test. Statistical significance analysis was carried out using a post hoc Mann–Whitney test. *p* < 0.05 was set as the significant level between each group.

## 4. Conclusions

Here, for the first time, we report that anti-oxidant c-Phycocyanin functionalized AgcPCNP promotes fibroblast cell migration, which was confirmed by a versatile approach of the signaling pathways mediated gene array. Additionally, the successful amalgamation of fluctuated marker genes in control, AgNP and AgcPC groups was further tested by using specific inhibitors for NFĸB (Nfi) and PI3K (LY294002). Studies indicated the importance of these two pathways in dermal wound healing. The study provides a novel insight into the beneficial aspects of metal conjugated cPC in the regulation of fibroblast cell migration, which is principally governed through the NFĸB pathway route but less significantly through the PI3K pathway. The functional validation of the importance of the NFĸB pathway was revealed by in vitro wound closure studies performed in the presence of an NFĸB inhibitor.

## Figures and Tables

**Table 1:** Signaling pathway and respective marker genes in mouse signal transduction pathway finder array during cell development, proliferation and migration.

Pathway	Marker Gene
Mitogenic pathway	*Egr1, Fos, Jun, Nab2*
WNT pathway	*Birc5, Cend1, Cdh1, Fgf4, Jun, Lef1, Myc, Pparg, Tcf7, Vegfa, Wisp1*
Hedgehog pathway	*Bmp2, Bmp4, En1, Foxa2, Hhip, Ptch1*
TFGβ pathway	*Cdkn 1a, Cdkn 1b, Cdkn 2a, Cdkn 2b*
JNK pathway	*Bcl2, Bcl 211*
P53 pathway	*Bax, Cdkn1a, Ei24, Fas, Gadd 45a, Igfbp3, Mdm2*
Stress signal pathway	*Atf2, Fos, Hsf1, Hspb1, Myc, Trp53*
NFAT pathway	*Cd5, Fas1*
CREB pathway	*Cyp19a1, Egr1, Fos*
JAK/STAT pathway	*Cxcl9, Li4ra, Irf1, Mmp10, Nos2*
Estrogen regulation pathway	*Bcl2, Brca1, Grb1, Igfbp4, Nrip1*
Androgen regulation pathway	*Cdk2, cdkn 1a*
Ca^2+^-Protein Kinase C pathway	*Csf2, Fos, II2ra, Jun, Myc, Odc1, Tfrc*
Phospholipase C pathway	*Bcl2, Egr1, Fos, Icam1, Jun, Nos2, Ptgs2, Vcam1*
Insulin regulation pathway	*Cebpb, Fasn, Gys1, Hk2, Lep*
LDL pathway	*Ccl2, Csf2, Sele, Vcam1*
Retinoic acid regulation pathway	*En1, Hoxa1, Rbpn1*
PI3K pathway	*Bcl2, Ccnd1, Fnf1, Jun, Mmp7, Myc*
NFĸB pathway	*Ccl20, Icam1, Ikbĸβ, Lep, NFĸb1a, Nos2, Tnf, Vcam1*

**Table 2:** Marker gene fluctuations in Mitogenic, Wnt, Hedgehog, TGFβ, JNK, p53 pathways during control, AgNP and AgcPCNP treated group.

Gene Name	AgNP	AgcPCNP	cPC
Mitogenic pathway genes			
*Egr1*	9.4657	6.3892	7.3787
*Fos*	4.5002	1.3673	3.6734
*Jun*	1.7291	2.0034	1.7854
*Nab2*	1.3665	−2.2763	−1.3673
Wnt signaling pathway genes			
*Birc5*	−2.20783	−1.43904	0.00934
*Cend1*	−1.7532	−2.2605	−2.0094
*Cdh1*	−4.3169	−6.75623	−6.0934
*Fgf4*	−4.7632	5.5281	−39095
*Jun*	1.7236	2.0465	1.9734
*Lef1*	1.5911	1.7092	1.5763
*Myc*	−1.3864	1.3887	1.3872
*Pparg*	4.6873	3.8956	2.8945
*Tcf7*	2.0345	−7453	1.9835
*Vegfa*	1.38874	−1.5463	−1.8934
*Wisp1*	1.7843	1.3288	1.4352
Hedgehog signaling pathway genes			
*Bmp2*	2.9032	−19834	1.9645
*Bmp4*	−1.3762	1.8932	−1.4763
*En1*	−1.7834	−1.4782	−1.9034
*Foxa2*	−16.354	−14.3784	−12.9834
*Hhip*	1.6723	1.2328	1.2673
*Ptch1*	1.3723	−1.2673	1.9453
TGFβ pathway genes			
*Cdkn 1a*	−1.2664	−1.3784	0.9945
*Cdkn 1b*	−9.3623	−8.4872	−7.3725
*Cdkn 2a*	−1.3765	−1.4783	−1.3744
*Cdkn 2b*	−1.3784	−1.3674	1.3364
JNK pathway genes			
*Bcl2*	−1.7634	−1.9983	−1.2783
*Bcl211*	2.4563	1.8964	2.3321
p53 pathway genes			
*Bax*	−1.3564	−1.8873	−1.2234
*Cdkn 1a*	−1.0734	−1.8435	−1.0034
*Ei24*	−1.6873	−1.7763	−1.5342
*Fas*	−3.8734	−1.2763	−2.8934
*Gadd45a*	1.6723	1.6624	1.3784
*Igfbp3*	1.6724	1.7724	1.0935
*Mdm2*	2.9835	2.0934	1.2753

**Table 3:** Array of marker gene fluctuations in stress, NFAT, CREB, JAK-STAT, Estrogen, Androgen and Ca^2+^ protein kinase pathways.

Gene	AgNP	AgcPCNP	cPC
Stress marker genes			
*Atf 2*	10.6723	9.3452	10.2674
*Fos*	4.2754	1.8342	3.9834
*Hsf1*	−1.3764	−1.8832	0.0037
*Hspb1*	28.3642	21.2732	19.3674
*Myc*	−1.2534	−1.1432	1.2874
*Trp53*	1.3426	−1.2653	1.2784
NFAT signaling pathway genes			
*Cd5*	−1.0263	−1.0924	−1.1163
*Fas1*	1.0564	−1.2734	1.0932
CREB pathway genes			
*Cyp19a1*	2.7634	2.9834	1.8934
*Egr1*	9.2634	6.2673	7.1863
*Fos*	3.9834	4.0152	3.6734
JAK-STAT signaling pathway genes			
*Cxcl19*	−1.2654	−2.0934	−2.6143
*Li4ra*	7.3562	7.2263	6.7834
*Irf1*	−1.2734	−1.0034	−1.3564
*Mmp10*	2.5643	2.456	1.9845
*Nos2*	1.7834	1.4563	−0.8934
Estrogen regulator pathway genes			
*Bcl2*	−1.3674	−1.5432	−1.3763
*Brca1*	1.0342	1.3654	−1.3674
*Grb1*	1.3452	1.2218	1.1783
*Igfbp4*	−4.1923	−3.8934	−4.0032
*Nrip1*	1.6734	1.1178	1.2784
Androgen regulatory pathway genes			
*Cdk2*	−1.3564	−1.0094	−1.3564
*Cdkn1a*	−1.2754	−1.4675	−1.0034
Ca^2+^ protein kinase pathway genes			
*Csf2*	3.2613	2.9843	3.1178
*Fos*	4.1173	3.9845	4.1342
*II2ra*	1.7832	1.1563	1.2673
*Jun*	1.7342	1.5632	1.2343
*Myc*	−1.1783	−1.0035	−1.5623
*Odc1*	2.3413	1.7834	2.2173
*Tfrc*	2.2034	2.6723	1.8934

**Table 4:** Fluctuations of marker genes in different signaling pathways such as Phospolipase, Insulin, LDL and Retinoic acid regulation pathways during different treatments as described in the methods.

Gene	AgNP	AgcPCNP	cPC
Phospolipase C signaling pathway genes			
*Bcl2*	−1.8404	1.2564	−1.00934
*Egr1*	9.3564	8.8934	6.8435
*Fos*	4.2673	4.8913	3.9803
*Icam1*	−1.3673	−2.09245	−1.8923
*Jun*	1.5632	1.0934	1.0023
*Nos2*	1.4742	1.8723	1.0923
*Ptgs2*	14.2262	13.0934	12.8723
*Vcam1*	−2.0934	−2.9834	−1.0934
Insulin regulatory pathway genes			
*Cebpb*	1.5672	1.8934	1.0925
*Fasn*	1.2893	1.2229	1.1093
*Gys1*	1.18923	1.9023	1.8992
*Hk2*	−1.2893	0.0943	−1.9283
*Lep*	1.4044	1.3984	1.29984
LDL pathway genes			
*Ccl2*	−3.2854	−2.9453	−3.4983
*Csf2*	3.1903	3.0021	2.9834
*Sele*	3.6724	3.9913	3.0932
*Vcam1*	−2.2658	−2.2093	−1.0046
Retinoic acid regulatory pathway genes			
*En1*	−1.5623	−1.8934	−1.0925
*Hoxa1*	1.0425	1.8723	1.0936
*Rbpn1*	4.724	3.7931	3.9982

## Data Availability

Data sharing not applicable.

## Abbreviations

AgNP	Silver nanoparticle
AgcPCNPs	C-phycocyanin conjugated silver nanoparticles
FRAC	Ferric Reducing Anti-oxidant power
ABTS	2,2′-azino-bis (3-ethylbenzothiazoline-6-sulfonic acid)
HSBS	Hank’s Balanced Salt Solution
FBS	Fetal Bovine Serum
PBS	Phosphate buffered solution
ICPS	Integrated Coupled Plasma Emission Spectroscopy
Nfi	NFĸB inhibitor
LY294002	PI3K inhibitor
cPC	c-Phycocyanin
